# XplorSeq: A software environment for integrated management and phylogenetic analysis of metagenomic sequence data

**DOI:** 10.1186/1471-2105-9-420

**Published:** 2008-10-07

**Authors:** Daniel N Frank

**Affiliations:** 1 Department of Molecular, Cellular, and Developmental Biology, Mucosal and Vaccine Research Program Colorado, University of Colorado, Boulder, CO, USA 80309, USA

## Abstract

**Background:**

Advances in automated DNA sequencing technology have accelerated the generation of metagenomic DNA sequences, especially environmental ribosomal RNA gene (rDNA) sequences. As the scale of rDNA-based studies of microbial ecology has expanded, need has arisen for software that is capable of managing, annotating, and analyzing the plethora of diverse data accumulated in these projects.

**Results:**

XplorSeq is a software package that facilitates the compilation, management and phylogenetic analysis of DNA sequences. XplorSeq was developed for, but is not limited to, high-throughput analysis of environmental rRNA gene sequences. XplorSeq integrates and extends several commonly used UNIX-based analysis tools by use of a Macintosh OS-X-based graphical user interface (GUI). Through this GUI, users may perform basic sequence import and assembly steps (base-calling, vector/primer trimming, contig assembly), perform BLAST (Basic Local Alignment and Search Tool; [1-3]) searches of NCBI and local databases, create multiple sequence alignments, build phylogenetic trees, assemble Operational Taxonomic Units, estimate biodiversity indices, and summarize data in a variety of formats. Furthermore, sequences may be annotated with user-specified meta-data, which then can be used to sort data and organize analyses and reports. A document-based architecture permits parallel analysis of sequence data from multiple clones or amplicons, with sequences and other data stored in a single file.

**Conclusion:**

XplorSeq should benefit researchers who are engaged in analyses of environmental sequence data, especially those with little experience using bioinformatics software. Although XplorSeq was developed for management of rDNA sequence data, it can be applied to most any sequencing project. The application is available free of charge for non-commercial use at .

## Background

The recent explosions in culture-independent studies of environmental DNA sequences ("metagenomics") and automated DNA sequencing capabilities have prompted the creation of numerous software applications designed to aid the analysis of an avalanche of sequence data. However, many of the commonly used, freely available applications require some facility with the UNIX/Linux operating system and/or specialized scripting languages to either manipulate files in batch or pipe data between applications. As automated DNA sequencing and sequence analysis has become commonplace in laboratories that do not specialize in bioinformatics, need has arisen for the development of powerful, yet simple-to-use, software.

**Figure 1:**
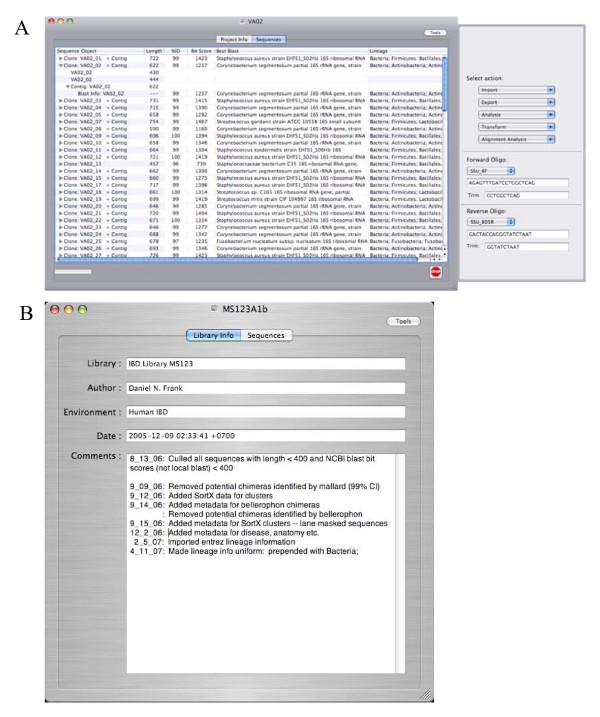
**Main XplorSeq window**. Screen shots of XplorSeq main window. (A) Listing of imported sequences, contigs, and blast data. Import, export, data analysis, and data transformation options are presented in menus within the tool drawer adjacent to the main window. (B) Project-associated data fields. Comments box provides space for recording details of analysis.

XplorSeq, written for the Macintosh OS X operating system, provides a graphical user interface (GUI) that integrates the use of several popular UNIX-based DNA sequence analysis applications. A number of additional features have been incorporated in order to track, annotate, and analyze sequence information in a manner conducive to high-throughput metagenomics. Implementation of a common GUI (Fig. 1) eliminates the need for the user to possess the kind of knowledge generally restricted to bioinformaticists and computer professionals: UNIX/Linux command shells (tsh, csh, or bash) and associated scripting languages. By presenting a unified GUI, XplorSeq, simplifies sequence analysis projects by allowing the user to focus on science, rather than the details of disparate software.

XplorSeq was developed for rapid compilation and analysis of rDNA clone libraries, but should be applicable to any sequencing project (computer hardware may, however, limit the scale of projects). Although several commercial and non-commercial software packages implement some of the same basic functionalities as XplorSeq, the development of XplorSeq was motivated by the absence of GUI-based software designed specifically for high-throughput, batch analysis of rDNA sequences, such as arise from culture-independent metagenomic studies. Specifically, the extant software could not accommodate the phylogenetic orientation of analyses and sequence annotations that are most useful for metagenomics. In contrast, XplorSeq implements several domain-specific software tools (e.g. for state of the art phylogenetic tree inference, OTU clustering, biodiversity estimates) that are not available in general-purpose DNA analysis packages. Many published studies, from a variety of laboratories engaged in metagenomics, have used XplorSeq, and thereby established its stability, ease-of-use, and capabilities [4-29]. The software is freely available for non-commercial use at .

## Implementation

XplorSeq is written in Objective-C using the Cocoa application framework (Apple Inc.). Releases are compiled for the OS X operating system (current versions require OS 10.4.x or 10.5.x) as universal binaries, which run natively on Macintosh computers with Intel or PowerPC microprocessors. Similar to Cocoa, the architecture of XplorSeq is based on the Model-View-Controller (MVC) design pattern. XplorSeq is multi-threaded and can adjust its operation to accommodate multiple shared-memory microprocessors.

The rationale for implementing XplorSeq as a standalone Macintosh application involved 1) desire for a highly responsive, feature-rich graphical output, thus precluding web-based applications; 2) recognition that the BSD-Unix operating system underpinning OS X would allow leveraging of existing open-source software; 3) observation that many computer novices (an intended audience for this software) were more comfortable with OS X than other operating systems; and 4) the maturity, stability, and support inherent in the Cocoa application framework.

Third-party software packages and plugin executables (sortx and biodiv) were written in C and C++. When possible, compiled executables are incorporated directly into the XplorSeq application bundle (essentially, a hidden directory structure) so that users can install and operate XplorSeq without the need for local compilation or extensive configuration. Full implementation of XplorSeq requires separate installation of phred and phrap (obtained at ).

## Results and discussion

The following sections outline the data structures and analytic tools that form the basis of the XplorSeq workflow.

### Data organization and GUI architecture

XplorSeq uses a document-based approach for project data management in which multiple sequences and their associated data are stored and accessed in a single file. As a project evolves, sequences may be added, deleted, amended and analyzed as needed. XplorSeq does not enforce a highly constrained analytic schema and thereby grants the user more autonomy in designing and implementing an analysis plan than typically is possible in a hard-wired software pipeline.

**Figure 2:**
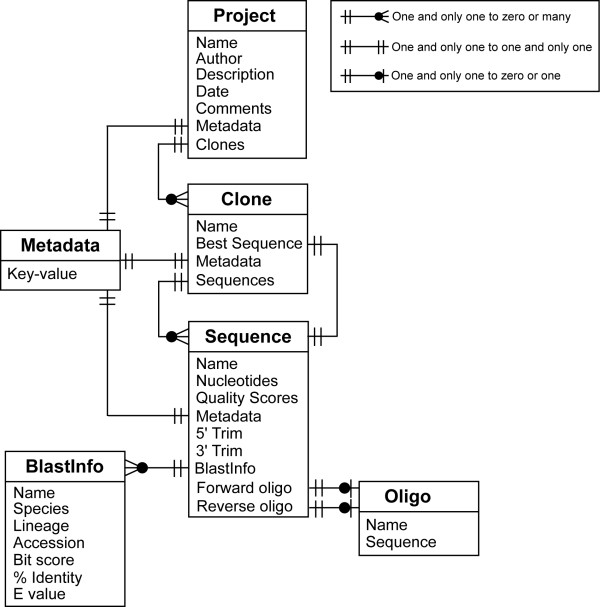
Organization of basic XplorSeq Data structures.

Data are organized as a hierarchy of data objects (Fig 2), which represent the model layer in XplorSeq's Model-View-Controller design pattern architecture. Each data object class (e.g. Sequence, Project) is associated with a window (the view layer) that is used to inspect data associated with particular instances of the class.

The top layer data object is the "Project", which stores all other data and is synonymous with the document as a whole. Hence, the main XplorSeq window (Fig 1) is the Project Inspector window. Projects organize and manage lists of "Clones", which represent individual cloned genes or PCR amplicons. Clones, in turn, manage groups of "Sequences" which map to unique DNA sequences. Sequences can be imported directly (e.g. as polished GenBank sequences), read from DNA sequencer traces, or assembled from other sequence objects ("contigs"). For each sequence analyzed by BLAST, XplorSeq creates a "BlastInfo" object that summarizes pertinent blast output data: identity and phylogenetic lineage of the sequence's closest BLAST hit, BLAST statistics, etc. Each Clone ranks its constituent Sequence objects based on BLAST bit-score and the "Best Sequence" (i.e. that with the highest bit-score) serves as a proxy for the entire Clone. "Oligo" objects encapsulate data that describe oligonucleotide sequences used in construction of clone libraries.

**Figure 3:**
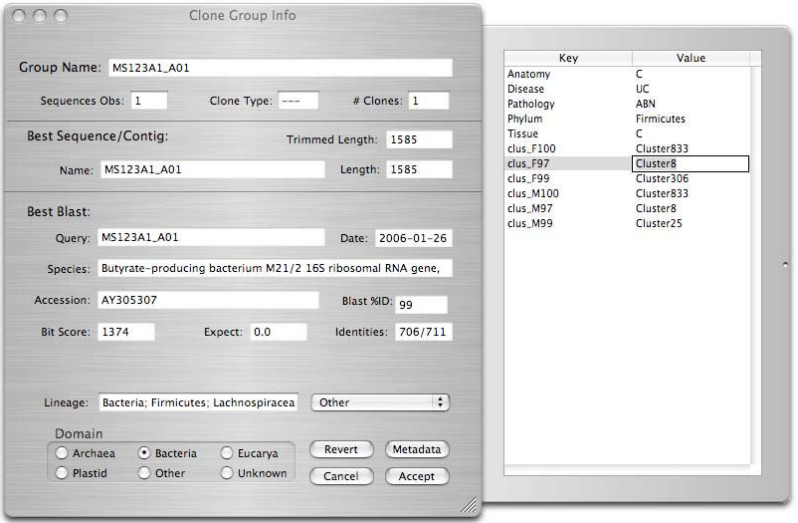
**Clone inspector window**. Summarizes information associated with a selected clone, a group of sequences (both individual reads and contigs) from the same amplified or cloned gene. The main window summarizes the top BLAST hit for the clone (i.e. the sequence or contig BLAST hit with the highest bit-score). The phylogenetic lineage of the clone can be assigned at the bottom of the main window. The drawer to the right of the window presents the contents of the meta-data dictionary associated with this clone.

**Figure 4:**
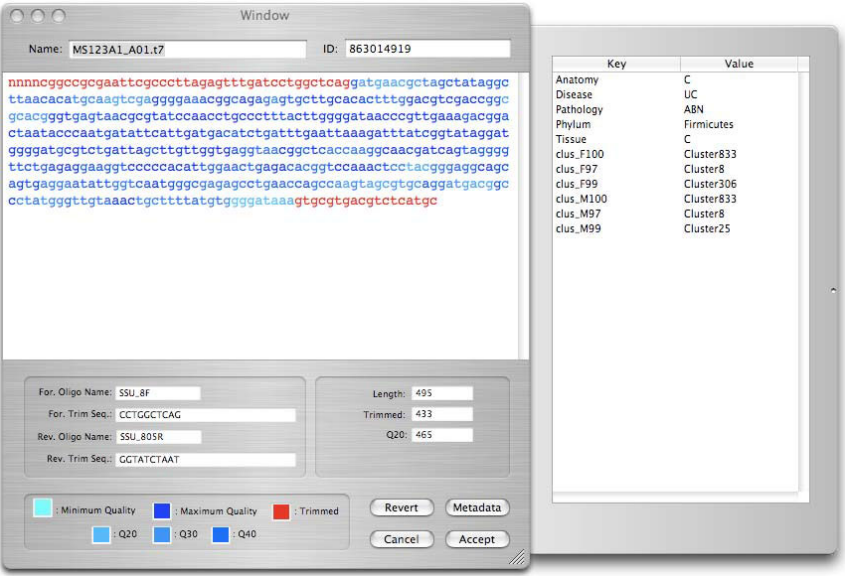
**Sequence inspector window**. Displays information associated with a selected sequence. Nucleotides are color-coded to represent the quality scores of individual nucleotides (the legend at the bottom of the window shows the meaning of the colors). Primers used to amplify the gene are summarized on the lower left. Basic summary statistics (length, trimmed length, and number of nts > Q20) are presented in the lower right. The drawer to the right of the window presents the contents of the meta-data dictionary associated with this sequence.

**Figure 5:**
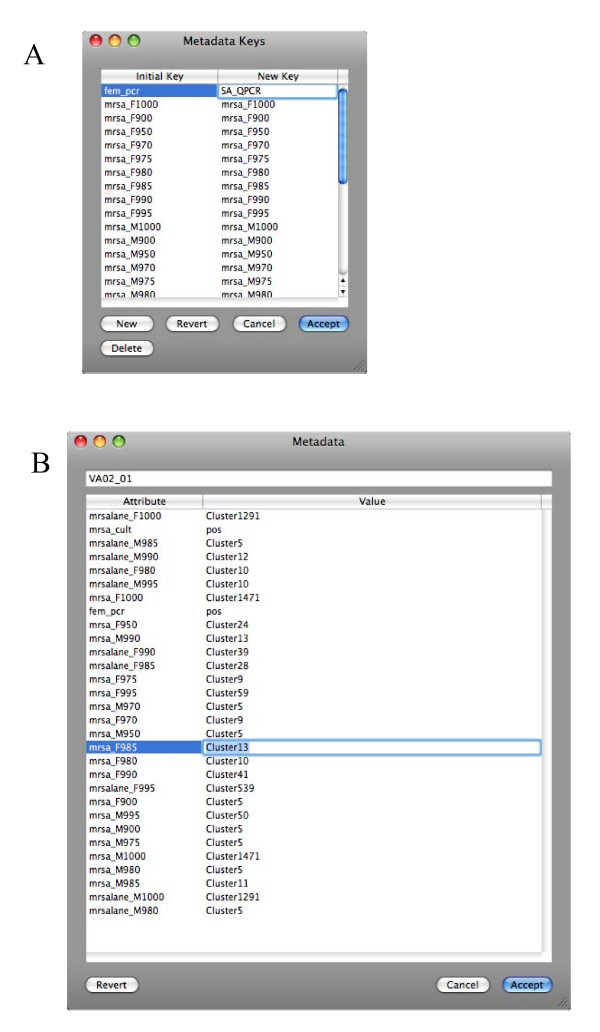
**Metadata editors**. A) Editing all keys for meta-data in project. B) Editing values associated with keys for a particular sequence object.

One of XplorSeq's more important design goals is to provide users with an extremely flexible and simple means of annotating data objects with meta-data consisting of user-specified information. XplorSeq implements meta-data through two strategies: 1) hard-coded data objects (i.e. "BlastInfo" and "Oligo" objects) and 2) a customizable "Metadata" object. Metadata objects are implemented as key-value dictionaries (e.g. hashes, maps) that can be linked to Project, Clone, and Sequence data objects. Users specify keys and values for particular objects through import of tab-delimited spreadsheets in which the first column designates the names of objects (i.e. sequence/clone names) and subsequent columns specify values (keyed by column headings) to attach to the objects. Alternatively, object inspector windows provide GUI-based features in which to display and edit meta-data (c.f., Figs. 3, 4, and 5).

### Data display and control of data processing

A project's Clone, Sequence, and BlastInfo objects are displayed in the Project Inspector window (Fig. 1A), which functions as the main XplorSeq window. Data are arranged hierarchically to reflect nesting of data structures. For each Clone, a summary of its best blast hit, which includes the taxa name, percent sequence identity and bit-score is displayed in the main XplorSeq window. The phylogenetic lineage of the top blast hit can be imported (through either an entrez idfetch query or import of tab-delimited data) to provide information about the taxonomic placement of a clone.

The user controls all steps of data processing by selecting objects to be acted upon and then choosing a function from menu items presented in the tool panel that extends from the main XplorSeq window (Fig 1A). Methods to import, export, and analyze data are accessible through these menus. Below the menus lie controls through which oligonucleotides used to generate PCR libraries can be designated, if relevant to the project; entries in the oligo menus can be modified through a preferences dialog.

Project specific meta-data can be recorded in several text fields presented in the Project window, under the "Project Info" tab. An editable text box is presented in which the user can enter comments, for instance details specific to a project (Fig 1B).

By double-clicking on an entry in the Project window, the user can display and edit more detailed information associated with that entry. For example, the Clone Inspector window (Fig. 3) summarizes the content of the Clone Object, including its top BLAST hit sequence and corresponding BlastInfo Object. The phylogenetic lineage and domain of the Clone Object can be set through the controls at the bottom of the window. A panel extending from the Clone Inspector window presents user-specified meta-data associated with the Clone Object.

DNA sequences, including contig sequences, are displayed in a Sequence Inspector window (Fig. 4). Individual nucleotides are color-coded to represent quality scores generated by the base-calling software (shades of blue) or trimmed sequences (red). Basic sequence information, such as primer sequences and trimmed sequence length, is displayed in a set of text fields at the bottom of the Sequence Inspector window. Similar to Clone Objects, sequence specific meta-data can be viewed through a panel that extends from the inspector window.

### Tools for data import and analysis

Table 1 summarizes the key functions available through XplorSeq. A user can perform base-calling on chromatograms generated by automated DNA sequencers, (through tracetuner [30] or phred, [31,32]); trim away vector, primer, and poor quality sequences; assemble contiguous sequences (TIGR_Assembler [33] or phrap, [31]); search for homologous sequences in sequence databases (blastcl3 or blastall; [1-3]); format BLAST databases (formatdb), perform multiple sequence alignments (clustalW; [34-36]); define Operational Taxonomic Units (sortx; D.N. Frank, unpublished); calculate distance matrices (phylip dnadist [37]); construct phylogenetic trees (clearcut, [38]; phylip neighbor, [37]; RAxML, [39]); and calculate a variety of biodiversity indices (biodiv; D.N. Frank, unpublished). By integrating software tools into a single application, XplorSeq facilitates seamless workflow through the sequence analysis process. Many of the key analytic steps are piped together, thus automating data processing. All data analysis operations may be executed in batch so that multiple sequences can be processed with minimal user intervention.

**Table 1:** Summary of XplorSeq functionality

**Function**	**Description**^**1**^
**Import**	
Chromatogram...	Import DNA chromatograms (.esd, .scf, .abi etc.): phred
PHD...	Import DNA sequences in Phd format
Contig...	Import DNA sequences and quality scores in FastA format
Blast...	Parse Blast records
FastA...	Import DNA sequences in FastA format
XplorSeq Library...	Import XplorSeq document
Phylogenetic Lineage...	Import phylogenetic lineage information from entrez
Metadata...	Import metadata in key-value format

**Export**	
Sequences...	Export DNA sequences in variety of formats
FastA + Qual...	Export DNA sequences and quality scores
Blast Info...	Export summary of Blast records
Cluster Table...	Enumerate OTUs belonging to groups of sequences
OTU Diversity...	Calculate OTU richness for set of sequences
Quality Scores...	Export summary of quality scores
Blast Accession #'s...	Export accession numbers of top Blast hits
Sequin Script...	Export data in format for Genbank submission (sequin)
Blast Database...	Create a Blast database (formatdb)
XML File...	Export data in XML format
Metadata...	Summarize and export metadata
Placeholder Tree...	List selected sequences in Newick format

**Analyze**	
Basecall->Blast...	Pipe data from chromatogram through Blast analysis
Contig->Blast...	Pipe data from contig assembly to Blast analysis
Basecall...	Perform base calling (phred or ttuner)
Contig...	Perform contig assembly (phrap or TIGR_Assembler)
Blast NCBI...	Blast query of Genbank
Blast Local...	Blast query of local blast database
Get Entrez Lineage Info.	Download entrez phylogenetic lineage information (idfetch)
Align...	Perform multiple sequence alignment (clustal)
Biodiversity (biodiv)...	Calculates biodiversity indices with random resampling (biodiv)
XplorSeq Doc Difference...	Generate differences between two XplorSeq documents

**Transform**	
Edit Sequence Names...	Alter names of sequences
Edit Lineage Names...	Edit phylogenetic lineage information
Edit Metadata...	Edit metadata associated with sequence
Edit Metadata Keys...	Edit all metadata keys in document
Group...	Group sequences and contigs
UnGroup...	Ungroup sequences and contigs
Clean...	Delete blast information, contigs
Sort...	Sort records in document
Set Oligos...	Associate primer sequences with sequence objects
Trim...	Trim sequences based on quality score and primer
UnTrim.	Remove trimming information
Rev.-Complement	Reverse complement sequence
DNA -> RNA	Convert DNA sequence to RNA sequence
RNA -> DNA	Convert RNA sequence to DNA sequence
UPPER CASE	Convert sequence to upper case
lower case	Convert sequence to lower case

**Alignment Analysis**	
OTU clustering	Cluster Operational Taxonomic Units (sortx)
Clearcut NJ Tree...	Fast neighbor joining trees (clearcut)
Phylip distance matrix...	Calculate distance matrix (dnadist)
Phylip NJ Tree...	Calculate Neighbor joining or UPGMA trees (neighbor)
Phylip seqboot...	Generate bootstrap replicates of alignment (seqboot)
Phylip consense...	Generate consensus of multiple trees (consense)
RAxML...	Generate Maximum Likelihood tree (raxmlHPC)

^1^Command line executables are listed in parentheses.

XplorSeq facilitates batch BLAST [1-3] analyses of DNA sequences through both networked and local searches of nucleotide databases. Local BLAST searches require properly formatted sequence databases, which may be downloaded from NCBI  or created by use of the formatdb executable (through XplorSeq or the command line). XplorSeq dispatches sequences to the appropriate client software (blastcl3 or blastall; [1-3]) and then parses the resulting output file into BlastInfo Objects.

**Figure 6:**
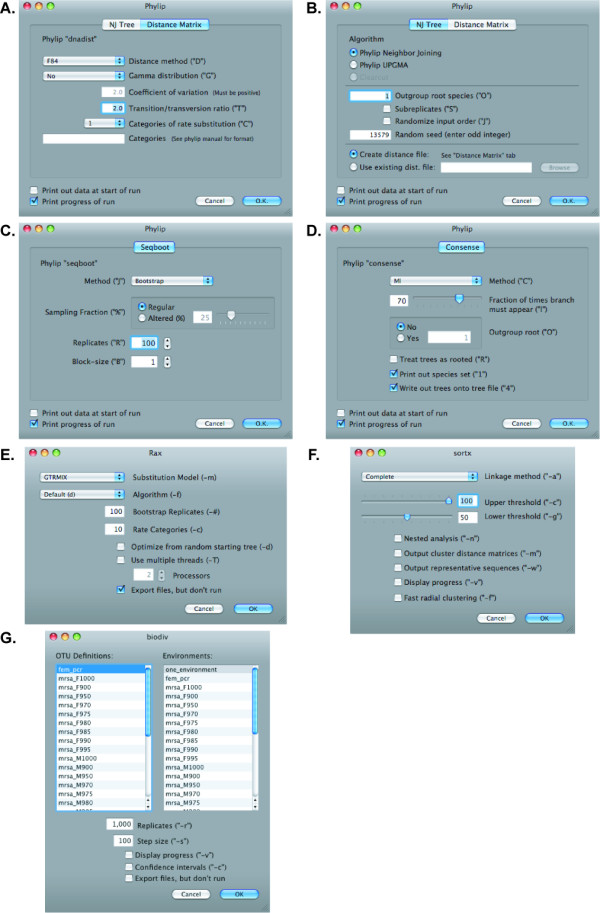
**Analyses of aligned sequences**. XplorSeq provides GUI-based access to several command-line programs used for phylogenetic analysis of multiple sequence alignments, including A-D) several commonly used programs from the phylip package [37]; E) RAxML for maximum-likelihood phylogenetic inference; F) sortx, for rapid clustering of sequences into OTUs; and G) biodiv for estimation of biodiversity indices through resampling statistics.

XplorSeq provides graphical interfaces for several commonly used command-line phylogenetic tools are shown in Fig. 6. Phylip modules for distance matrix calculation, neighbor-joining/UPGMA trees, data-set bootstrapping, and consensus tree generation are accessible through these interfaces [37]. Similarly, fast neighbor-joining and maximum-likelihood phylogenetic tree inference are available (clearcut [38] and RAxML [39], respectively). In each case, following selection of the appropriate options, sequence data are exported in the required format, the analysis performed, and results imported (if appropriate) into XplorSeq. For tasks that may take prolonged time to execute (e.g. RAxML [39]), a file listing the command-line invocation of the program is saved along with all data required for the task; these files can be transferred to another computer for subsequent execution of the scripted command.

OTU clustering is implemented through the program sortx, which was written in tandem with XplorSeq (Fig. 6F). Sortx uses a fast radial clustering algorithm to bin aligned sequences based on uncorrected pairwise sequence distances (%ID). Clusters can be assembled based on furthest-, mean-, or nearest-neighbor rules. Following cluster formation, sortx selects a representative sequence for each cluster, which maximizes both pairwise similarity to other cluster members and sequence length (simply choosing the sequence with minimum pairwise distance could select for short, but well-conserved sequences, which would not necessarily be representative of the cluster). Finally, the user can select a range of pairwise sequence distance thresholds by which to assemble OTUs in order to create multiple data sets at different phylogenetic depths.

Estimates of biodiversity indices (species richness, diversity, evenness) can be reported through either of two modes. First, the export function OTU Diversity... reports basic calculations of commonly used indices (S_obs_, S_chao1_, C_ACE_, Good's coverage, Shannon diversity; [40]) for a set of selected sequences. Alternatively, the same biodiversity estimates can be made in a more thorough manner through execution of the analysis function Biodiversity (biodiv)..., which invokes the program biodiv, a standalone command-line tool built in conjunction with XplorSeq. As shown in Fig. 6G, the user selects OTUs definitions for the selected sequences through choice of meta-data options. To compare indices between different groups of sequences, the user can also select multiple "environments" by which to differentiate the sequence subsets; biodiv then performs separate analyses for each designated environment. Biodiv performs random resampling of OTUs and calculates collector's curves and associated biodiversity indices as a function of sampling effort [41]. Biodiv also reports rarefied biodiversity indices, based on resampling, with 95% confidence intervals for each type of environment [41].

### Tools for data export and transformation

**Figure 7:**
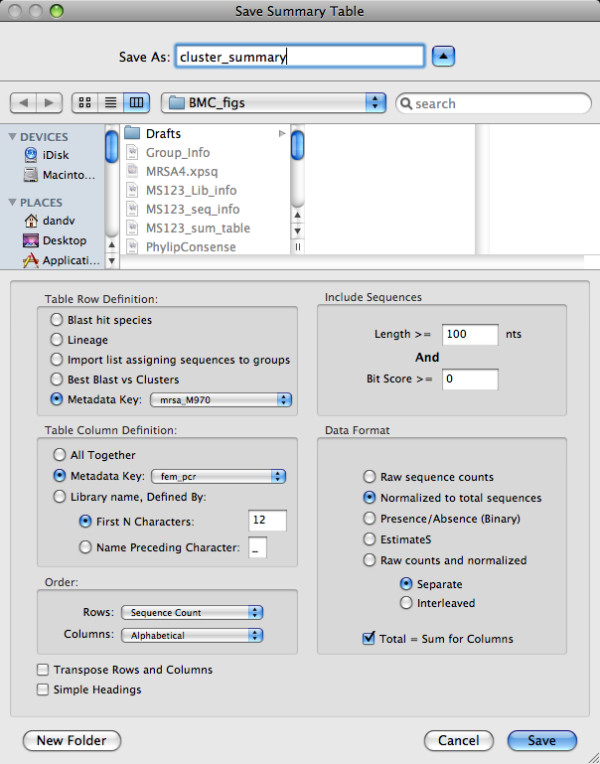
**Tabulation of sequence abundance/prevalence**. The Summary Table dialog provides multiple means of tabulating sequence data. In this example, rows are defined by the values of a meta-data key (e.g. 97% OTUs), while columns are defined by the value associated with another meta-data key (e.g. PCR results). The "Data Format" panel specifies that sequence counts in each column are normalized to the total of each column.

XplorSeq provides several features by which to export sequences and data for further analyses. Data reports are generated in tab-delimited format for import into spreadsheets. A particularly useful export feature tabulates sequence abundances and/or prevalences on the basis of user-defined terms that define how data are categorized and enumerated. For instance, as shown in Fig. 7, the user has opted to create a table in which rows are defined by meta-data that specify OTUs and columns are specified by meta-data that organize samples by the results of a PCR assay; alternatively, similar sequences can be grouped by BLAST results, or phylogenetic lineage. The cells of the output tables enumerate the abundance or presence/absence of sequences for a given row and column (Additional file 1).

**Figure 8:**
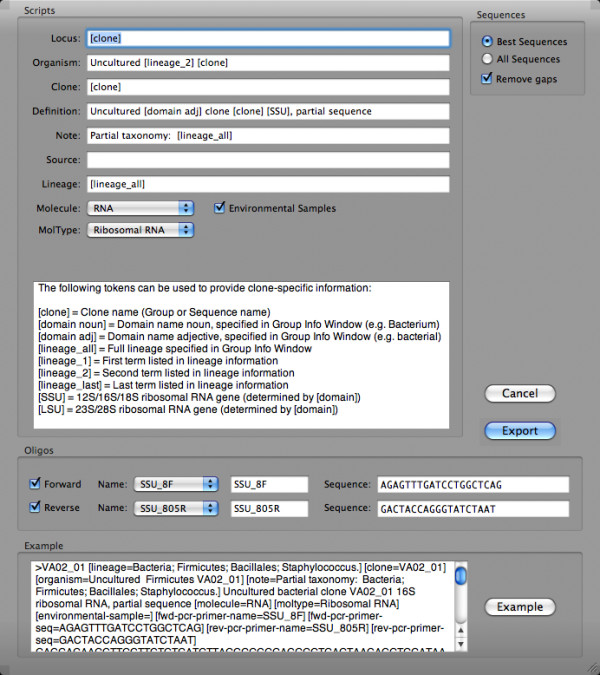
**Sequin script export**. Scripted export of sequence data for GenBank submission through Sequin. Data associated with each sequence can be manipulated in order to tailor the level of detail that will go into Sequin.

Preparation of sequence data for submission to public databases can be tedious and error-prone, despite the availability of tools such as NCBI's sequin software tool. XplorSeq can facilitate the submission process by automating the organization of sequences and their annotations into a form suitable for input into sequin (Fig. 8). The user can modify the output of the sequin script export function to include or exclude particular information and to tailor GenBank descriptors and/or features (e.g. Locus, Definition, Molecule fields).

**Figure 9:**
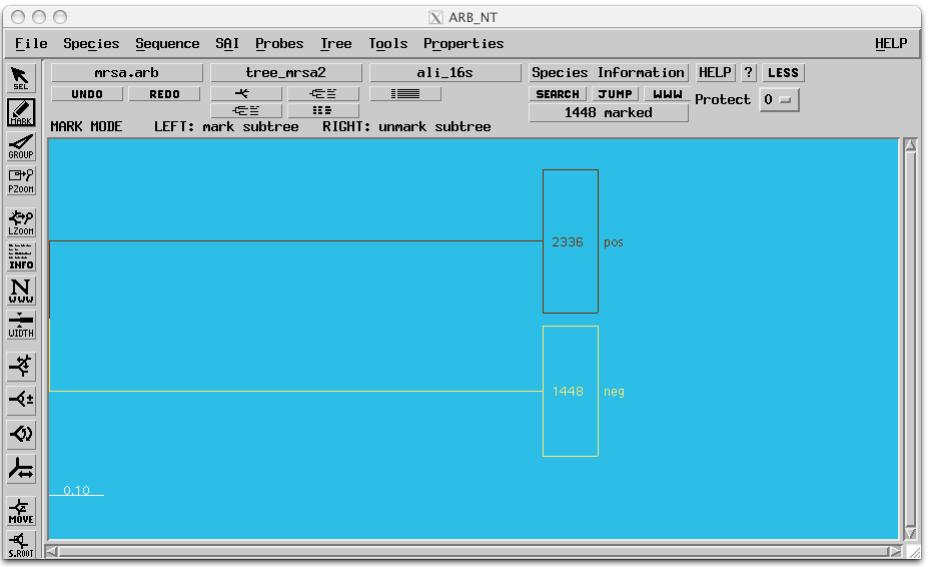
**Input of placeholder tree into ARB**. Sequences were exported from XplorSeq using the "Placeholder Tree" option. Sequences were split into two categories on the basis of associated meta-data (in this example, results of a PCR screen). Here, the user has selected all taxa belonging to the "neg" group. Because ARB propagates taxa markings between trees, placeholder trees can be used to graphically organize and manipulate groups of sequences that aren't necessarily related.

For detailed phylogenetic studies, sequences assembled by XplorSeq can be exported in formats suitable for input into other software packages, such as ARB [42], DOTUR [43], NAST [44], SILVA [45], or UniFrac [46]. Users can choose sequences to be exported by selecting particular objects or through filters for sequence length, bit-score, type of sequence (i.e. contig vs. raw sequence), or other meta-data. Finally, "placeholder" Newick-formatted phylogenetic trees can be exported in order to select specific subsets of sequences in tree visualization software such as ARB. In this format, taxa belonging to user-defined subsets (categorized, for instance, through specific meta-data) are placed in clades and assigned artificial branch-lengths of 0.1; inter-group distances are set to 0.9, which produces a well-demarcated organization of sequences into assigned groups. Import of such a tree into ARB allows rapid, graphical marking of different sets of taxa (Fig. 9), the results of which are propagated to other "real" trees in ARB.

### Performance issues

The data analysis tools employed by XplorSeq (e.g. phred, phrap, blast, sortx, biodiv) are invoked in separate threads, so that multiple tasks can be run in parallel. During these steps, feedback to the GUI is limited in order to minimize the overhead of performing analyses through XplorSeq. Typical latencies associated with XplorSeq contribute no more than an additional ~10% to the total elapsed time required for an analysis, relative to the same task performed through command line execution of the underlying 3^rd ^party software (Table 2). This overhead is due primarily to data import/export and parsing of results, which add value to the functionalities provided by command line utilities. Consequently, XplorSeq should not place any undue limitations on the use of command-line software to process and analyze sequence data, beyond those inherent in the computer system and/or software tool being used. In other words, tasks that can be performed on a particular Macintosh system through the command line should also be feasible through XplorSeq.

**Table 2:** Execution times of commonly used software: comparison of XplorSeq with command line implementation

	Execution Time (sec.)^1^					
	System A^2^		System B^3^			
Program	XplorSeq^4^	Command Line	XplorSeq^4^	Command Line	Task	Sequence Data
phred	50.5 (5.3)^1^	51.0 (2.9)^1^	23.3 (0.8)^1^	19.3 (0.5)^1^	Basecall	768 .esd files.
phrap	167.0 (2.4)	153.3 (0.8)	30.5 (1.2)	28.8 (1.5)	Contig	384 pairs of reads.
blastall	368.0 (2.4)	345.3 (0.8)	228.5 (1.2)	221.3 (2.4)	Local blast	24 1585-mers
XplorSeq	69.0 (8.1)	na	21.2 (0.4)	na	Import fasta	250,000 1585-mers
XplorSeq	130.8 (10.8)	na	12.2 (0.4)	na	Open XplorSeq file	250,000 1585-mers
XplorSeq	160.8 (14.4)	na	11.7 (0.5)	na	Save XplorSeq file	250,000 1585-mers
XplorSeq	60.5 (0.8)	na	46.3 (1.4)	na	Import fasta	1,000,000 25-mers
XplorSeq	216.3 (11.2)	na	28.0 (0.6)	na	Open XplorSeq file	1,000,000 25-mers
XplorSeq	34.3 (3.8)	na	16.5 (0.5)	na	Save XplorSeq file	1,000,000 25-mers

^1^Elapsed time of execution: Mean (St. Dev) seconds.

^2^Executed on a 2 GHz Intel Core Duo MacBook Pro. 1 GB 667 MHz DDR2 SDRAM. Mac OSX version 10.5.4.

^3^Executed on a workstation with 2 × 3 GHz Quad-Core Intel Xeon processors. 8 GB 800 MHz DDR2 FB-DIMM. Mac OS X Server version 10.5.4

^4^Elapsed time includes export and import of data.

The XplorSeq file format, implemented using the Cocoa software framework, does not significantly add to the size of data files. For example, an XplorSeq file containing an alignment of 250,000 rRNA sequences (1585 nucleotides per sequence) requires 396 megabytes (MB) of storage, compared to 381 MB for a fasta formated file storing the same alignment. The addition of metadata, such as blast results, increases file size in roughly linear proportion to the size of parsed input text.

Although the use of a flat file format by XplorSeq greatly simplifies data storage and transfer, it does require that all data be read into memory before being manipulated. For relatively large files, bottlenecks are apparent primarily in tasks requiring import and export of data. Thus, sequence analysis projects are likely to be limited as much by computer hardware (e.g., quantity of random access memory and bus speeds) and the performance of underlying 3^rd ^party software tools as by XplorSeq. However, the XplorSeq environment is scalable from laptops to more advanced workstations (e.g., 8-core/32 GB systems currently available) so its capabilities can be expanded as need arises and hardware evolves.

As a rough guide to system requirements, Table 2 presents benchmark comparisons of common XplorSeq tasks performed on a laptop and a workstation. To open the 250,000 sequence XplorSeq alignment file described above, for example, requires ~130 or ~12 seconds, on the laptop or workstation, respectively. Test datasets of 1 × 10^6 ^25-nucleotide sequences can be manipulated with similar lag times. More fully annotated files containing ~50,000 16S rRNA sequences, each of length ~1000 nucleotides, have been used routinely on a laptop with little performance degradation [17,23,27]. However, projects with > 50,000 annotated sequences likely will require higher performance workstations to insure the responsiveness customary to users of GUI-based software. Alternatively, sequence data can be spread across multiple files, each of which maintains information generated from a particular experiment (e.g. a PCR amplicon library). More complex data storage strategies, such as the use of application-specific databases may be implemented in the future if they do not compromise the XplorSeq philosophy of ease of software installation and use.

## Conclusion

Although XplorSeq was developed to expedite the phylogenetic analysis of ribosomal RNA (rRNA) gene libraries, it should prove useful in any sequencing project, particularly ones facilitated by batch analysis of multiple clones. Moreover, any UNIX-based DNA sequence analysis tool that can be ported to Mac OSX can be readily incorporated into XplorSeq. Suggestions for the addition of other modules to the XplorSeq package are most welcome.

## Availability and requirements

**Project name: **XplorSeq

**Project home page: **

**Operating system: **Macintosh OS X (currently requires 10.4.x or 10.5.x)

**Programming language: **Cocoa/Objective-C, C, C++

**Other requirements: **phred and phrap are available at 

**License: **Daniel N. Frank. Free for non-commercial use

**Any restrictions to use by non-academics: **Contact corresponding author. Users are requested to notify the corresponding author when XplorSeq is cited.

## Supplementary Material

Additional file 1 Example of sequence enumeration table. Click here for file
