# Supplementary material for: XplorSeq: A software environment for integrated management and phylogenetic analysis of metagenomic sequence data
Source: BMC Bioinformatics. 2008 Oct 7;9:420. doi: 10.1186/1471-2105-9-420 (PMC2577119; doi:10.1186/1471-2105-9-420)
Supplement: Additional file 1 — Example of sequence enumeration table. [file 1471-2105-9-420-S1.pdf]

Additional File. Example of sequence enumeration table.

mrsa_M970	Best Blast	Bit Score (mean)	Blast %ID (mean)	Prevalence pos	Prevalence neg	Domain	Lineage
Cluster121	Stenoxybacter acetivorans strain TAM-JN1 16S ribosomal RNA	569 - 771 (666)	85 - 92 (89)	0	56.99	Bacteria	Neisseriales;
Cluster16	Corynebacterium segmentosum partial 16S rRNA gene, strain	993 - 1398 (1286)	99	38.71	1.08	Bacteria	Actinomycetales;
Cluster5	Staphylococcus aureus strain EHFS1_S02Ha 16S ribosomal RNA	1265 - 1449 (1388)	99 - 100 (99)	22.58	9.68	Bacteria	Bacillales; Staphylococcus.
Cluster87	Pseudomonas putida strain ZX-PKU-004 16S ribosomal RNA	995 - 1088 (1031)	96 - 98 (97)	0	7.53	Bacteria	Pseudomonadales;
Cluster153	Actinobacillus pleuropneumoniae MCCM 00189 16S ribosomal	1328 - 1423 (1390)	98 - 99 (98)	0	7.53	Bacteria	Pasteurellales;
Cluster135	Streptococcus salivarius strain ATCC 7073 16S ribosomal	1263 - 1423 (1350)	98 - 100 (99)	6.45	0	Bacteria	Streptococcaceae;
Cluster81	Streptococcus mitis ATCC 903 16S ribosomal RNA gene,	1376 - 1435 (1403)	99	5.38	0	Bacteria	Streptococcaceae;
Cluster80	Streptococcus sp. oral strain 7A 16S ribosomal RNA gene,	1358 - 1437 (1405)	99	2.15	2.15	Bacteria	Streptococcaceae;
Cluster172	Staphylococcus lugdunensis isolate 13/06 16S ribosomal RNA	1376 - 1431 (1408)	99 - 100 (99)	0	3.23	Bacteria	Bacillales; Staphylococcus.
Cluster85	Pseudomonas sp. OCR2 gene for 16S rRNA, partial sequence.	1380 - 1423 (1401)	98 - 99 (98)	0	2.15	Bacteria	Pseudomonadales;
Cluster141	Rothia mucilaginosa 16S ribosomal RNA gene, partial	1231 - 1346 (1288)	98 - 99 (98)	2.15	0	Bacteria	Actinomycetales;
Cluster77	Staphylococcus aureus strain EHFS1_S02Ha 16S ribosomal RNA	1275 - 1404 (1339)	99	2.15	0	Bacteria	Bacillales; Staphylococcus.
Cluster139	Neisseria mucosa 16S rRNA gene (partial), strain LNP405.	1400 - 1405 (1402)	99 - 100 (99)	2.15	0	Bacteria	Neisseriales;
Cluster52	Peptoniphilus sp. WAL 10418 16S ribosomal RNA gene,	1209 - 1231 (1220)	96	0	2.15	Bacteria	Clostridia; Clostridiales;
Cluster86	Staphylococcus epidermidis 16S rRNA gene, clone 1V4.	1168	96	0	1.08	Bacteria	Bacillales; Staphylococcus.
Cluster88	Bacillus cereus strain GS03 16S ribosomal RNA gene,	1447	99	0	1.08	Bacteria	Bacillus; Bacillus
Cluster143	Actinomyces naeslundii 16S rRNA gene. <lineage>Bacteria;	1269	99	1.08	0	Bacteria	Actinomycetales;
Cluster148	Staphylococcus aureus strain EHFS1_S02Ha 16S ribosomal RNA	1049	98	1.08	0	Bacteria	Bacillales; Staphylococcus.
Cluster138	Corynebacterium segmentosum partial 16S rRNA gene, strain	1443	99	1.08	0	Bacteria	Actinomycetales;
Cluster149	C.striatum 16S rRNA gene (CIP 81.15T). <lineage>Bacteria;	1324	99	1.08	0	Bacteria	Actinomycetales;
Cluster78	Roseateles depolymerans strain 61A (DSM11813) gene for 16S	1231	98	0	1.08	Bacteria	Burkholderiales;
Cluster114	Anaerococcus vaginalis strain CCUG 31349 16S ribosomal RNA	898	92	0	1.08	Bacteria	Clostridia; Clostridiales;
Cluster162	Actinobacillus pleuropneumoniae MCCM 00189 16S ribosomal	1441	99	0	1.08	Bacteria	Pasteurellales;
Cluster147	Streptococcus mitis 16S rRNA gene, clone 2C4.	1053	94	1.08	0	Bacteria	Streptococcaceae;
Cluster136	Fusobacterium nucleatum subsp. nucleatum 16S ribosomal RNA	1235	97	1.08	0	Bacteria	Fusobacteriaceae;
Cluster142	Streptococcus genomosp. C3 16S ribosomal RNA gene, partial	1376	99	1.08	0	Bacteria	Streptococcaceae;
Cluster145	Corynebacterium segmentosum partial 16S rRNA gene, strain	1124	99	1.08	0	Bacteria	Actinomycetales;
Cluster152	Haemophilus parainfluenzae strain CIP 102513 16S ribosomal	1390	99	1.08	0	Bacteria	Pasteurellales;
Cluster150	Corynebacterium mucifaciens isolate 01-0118 16S ribosomal	1247	99	1.08	0	Bacteria	Actinomycetales;
Cluster137	Streptococcus mitis ATCC 903 16S ribosomal RNA gene,	1308	99	1.08	0	Bacteria	Streptococcaceae;
Cluster133	Streptococcus gordonii strain ATCC 10558 16S small subunit	1467	99	1.08	0	Bacteria	Streptococcaceae;
Cluster151	Granulicatella adiacens partial 16S rRNA gene, strain	1402	99	1.08	0	Bacteria	Carnobacteriaceae;
Cluster212	Beta proteobacterium ASRB1 16S ribosomal RNA gene, partial	1384	99	0	1.08	Bacteria	Betaproteobacteria.
Cluster144	C.striatum 16S rRNA gene (CIP 81.15T). <lineage>Bacteria;	1358	100	1.08	0	Bacteria	Actinomycetales;
Cluster146	Corynebacterium fastidiosum partial 16S rRNA gene, strain	938	99	1.08	0	Bacteria	Actinomycetales;
